# Perceptions of sexual risk behavior among Palestinian youth in the West Bank: a qualitative investigation

**DOI:** 10.1186/1471-2458-14-1213

**Published:** 2014-11-24

**Authors:** Salwa G Massad, Rita Karam, Ryan Brown, Peter Glick, Mohammed Shaheen, Sebastian Linnemayr, Umaiyeh Khammash

**Affiliations:** Palestinian National Institute of Public Health, Ramallah, Palestine; Juzoor for Health and Social Development, Ramallah, Palestine; RAND Corporation, Sta, Monica, USA; Al Quds University, Abu-Dis, Palestine; United Nations Relief and Works Agency for Palestine Refugees in the Near East (UNRWA), Jerusalem, Palestine

**Keywords:** Palestine, Youth, Sex, Behaviors, HIV

## Abstract

**Background:**

Young people in the Middle East and North Africa (MENA) are profoundly affected by violence, high unemployment, and economic hardship. Experiences of community-level violence and personal trauma increase the likelihood that young people will engage in risky behaviors that include smoking, drug use, and unsafe sex. Little is known about the sexual behavior of young people in the region, particularly in the occupied Palestinian territory (oPt). Our aim in this study was to gain an insight into the perceived prevalence and patterns of sexual behavior among Palestinian youth.

**Methods:**

The study was based on ten focus groups and 17 in-depth interviews with young people aged 16-24 years as part of the formative phase of a cross-sectional representative study of risk behaviors in the West Bank, including Jerusalem, in 2012. The sample was selected using a combination of purposive and convenience sampling. Qualitative analysis was used to code detailed notes of focus groups and interviews.

**Results:**

Based on participants’ reports, different types of sexual activity outside marriage were not uncommon, even in conservative communities. The most reported sexual activity was non-penetrative sex: oral and anal intercourse, and virtual sex. Some young people had sexual intercourse with sex workers; they went to brothels in Israel and to brothels operating clandestinely in the West Bank, including East Jerusalem. Most respondents were of the opinion that young people did not usually use protection during sexual intercourse. Many reported that youth engage in different types of sexual activity outside marriage for several reasons: to challenge the culture, financial constraints and inability to marry, basic human need, personal pleasure, suppression, to kill boredom, and to prove manhood.

**Conclusions:**

In contrast with the conservative social context of the occupied Palestinian territory (oPt), the findings suggest that sexual activities outside marriage may be more common than is currently assumed. Sexual behavior in the oPt is a concern because of the low awareness of the potential health consequences. The results draw attention to the need to incorporate sexual reproductive health into the national agenda and ensure that it is included in the programs of national institutions.

## Background

In the Middle East and North Africa (MENA) region, there has been a steady increase in incidences of unintended teenage pregnancies, sexually transmitted infections (STIs) and HIV infections, and gender based sexual violence [1]. After Eastern Europe and Central Asia, the MENA region has the fastest growing HIV/AIDS epidemic in the world, albeit from a very low base [2]. Available data reveal that STIs and HIV infections are more common among young people aged 15-29 years than among other age groups [3]. For these reasons, there is concern both that broader epidemics may soon emerge in MENA countries, and that youth are especially at risk of HIV and other sexually transmitted diseases [4, 5]. In addition, sex education in the MENA region is uncommon and is a controversial issue circumscribed by political, economic, cultural, and religious factors [3]. Societal taboos are major obstacles to informed discussions about sexual and reproductive health issues, particularly in relation to young people [3]. Very little is known about existing health risk behaviors among youth (or others) in the region, reflecting social strictures in addressing sensitive issues of sexual behavior, alongside the related perception on the part of some governments that HIV/AIDS will never become a serious problem in their countries.

Palestinian youth are profoundly affected by the Israeli occupation in a multitude of ways, including high unemployment and economic hardship, frequent humiliation or harassment, restrictions on movement, and long term exposure to violence at individual, family, and community level. Evidence from other contexts suggests that experiences of community-level violence and personal trauma increase the likelihood that young people will engage in risk behaviors, including unsafe sex or multiple partners, smoking and drug use [6, 7].

Despite the restrictions on travel between the Territories and Israel (and East Jerusalem), about 110,300 Palestinians from the West Bank continue to work in Israel and in Israeli settlements, while many thousands more are thought to enter illegally for work [8]. Young men under the age of 25 make up an unknown, but undoubtedly significant, proportion of these workers [9]. These movement patterns may constitute a major risk factor for HIV infection by increasing access to drugs and commercial sex, as well as potentially increasing the propensity to engage in risk behaviors when back home.

Although the HIV/AIDS epidemic is still at an early stage in Palestine, there are alarming signs that it is expanding [10]. HIV is mainly transmitted through heterosexual contact (56%), but in 5% of cases it is transmitted via blood products, 4% by sharing unsterilized needles for drug use, 4% mother-to-child, 4% bisexual, 2% homosexual (12% others, 13% unknown) [11]. Most HIV cases were of males and about 80% of HIV cases were in the 20–49 year age group [11]. As in the rest of the region, efforts to deal with rising concerns about HIV risks to young people are critically hampered by lack of data on most related risk behaviors among young Palestinian men and women. The specific aim of this qualitative research is to provide insights into the perceived prevalence and patterns of sexual behavior among Palestinian young men and women in the West Bank.

## Methods

As part of a larger study whose aim is to examine systematically the prevalence of sexual behavior among young Palestinians aged 16-24 in the West Bank and to understand the determinants of these behaviors, we conducted ten focus groups and 17 semi-structured individual interviews with young Palestinians in the West Bank. This paper presents the results from the study’s formative phase on the perceptions of youth to the prevalence of risk behaviors and the settings in which such behavior occurs. In the individual interviews, but not in the focus groups (FGs), participants were also asked to discuss their own behaviors. This study adheres to the Qualitative Research Review guidelines (RATS).

### Recruitment and sample

We utilized a combination of purposive and convenience sampling techniques to select a sample balanced across age, gender, region, and population density (villages, refugee camps, towns, and cities). We first selected communities from the southern, middle, and northern regions of the West Bank and East Jerusalem – including refugee camps and rural areas in some of those communities. Communities included Bethlehem, Hebron, Jericho, East Jerusalem, Kufr Aqab, Nablus, Ramallah, Shufat, Tulkarm, and Qalqylia. Recruitment was conducted by various organizations, including the Sharek Youth Organization, Juzoor for Health and Social Development, and United Nations Relief and Works Agency for Palestine Refugees in the Near East.

Five focus groups of young women and five focus groups of young men were conducted, with six to nine participants in each group. A total of 17 young adults (eight females and nine males) participated in individual interviews to explore in-depth issues related to their own behavior; nine of them were recruited from the focus groups. A total of 83 participants (42 males and 41 females) participated in the study. A demographic description of the study sample is reported in Table 1. Approximately 64% of the participants were students and 14% were in employment. With the exception of one young woman, all participants were single. As it was a convenient purposive sample, we aimed to have adequate representation from employed, unemployed, out of school, in school and college students, without necessarily reflecting the general youth population. In 2012, 43% of youth (15-29 years) were enrolled in education, school dropout rates were 31% [12], 33% of youth were unemployed [13], and 92% of females in the 15-29 year age group were married compared with 80% of males [12].

**Table 1:** **Demographic characteristics of participants (N = 83)**

Age		
Median		20
Range		16-24
	**N**	**%**
**Males**	42	50.6
**Geographical distribution**		
- North of West Bank	21	25.3
- Middle of West Bank	18	21.7
- South of West Bank	18	21.7
East Jerusalem	26	31.3
**Type of residency**		
- Urban	30	36.2
- Rural	26	31.3
- Camp	27	32.5
**Highest level of education**		
- Less than high school	21	25.3
- High school	10	12.0
- College	31	37.4
- University	21	25.3
**Employment status**		
- Employed	12	14.4
- Unemployed	20	24.2
- Student	51	61.4

### Focus group and interview procedures

The focus group and individual interview protocols explored the perceptions of young adults of the prevalence of health risk behaviors among their peers. Key questions included: sub-groups of youth who participate in sexual activities, type of sexual activities, locations where youth engage in sexual activities, protection used while engaging in sexual activities, reasons youth engage in sexual activities outside the frame of marriage, and perceived positive and negative consequences of engaging in sex outside marriage. The interviews also asked respondents about their own engagement in risk behaviors. Both protocols were semi-structured to ensure that key questions were addressed and to permit comparisons across individuals and groups, while at the same time, providing the interviewer with the freedom to follow up on unanticipated topics.

Interviewer gender was matched with the gender of the focus group or interview participants and all interviews were conducted in Arabic. The focus group interviews were audio recorded after obtaining the participants’ consent. Due to the fact that the individual interviews addressed sensitive personal behavior, these were not taped and the interviewer instead took detailed written notes. The RAND Corporation Ethics Committee, Santa Monica, CA and Al Quds University, Palestine, approved the study protocol for the formative research in January 2012. Oral informed consent and assent were obtained from participants and the parents of those aged below 18.

### Qualitative analysis

All notes were translated from Arabic into English. A sample of the translated interviews was reviewed against the Arabic version by research team members to ensure accuracy. Qualitative analysis was conducted on the English version of the notes using NVIVO version 9. Initial formal coding divided the data into characteristics of sexual behaviors and models explaining engagement in sexual activities.

Following accepted protocols for rigorous qualitative coding [14], each of these codes specified inclusion and exclusion criteria for the coding of excerpts, typical and atypical exemplars, and “close but no” examples. Within each of the formal codes referred to above, sub-codes were further developed to indicate discussions by sub-group, location, personal engagement in behaviors, and the like.

Data were coded by one of the three researchers situated in Palestine and two coders situated in the US reviewed and validated the coding. Any coding differences were discussed and resolved. We then summarized sub-coded text into bullet points with quotations, reporting the frequency of each sub-code by the number of focus groups and individual interviews.

## Results

### Characteristics of sexual behaviors

Most of the study participants perceived the engagement of young adults in sexual activities out of wedlock to be prevalent, despite living in a conservative society. Most believed that different kinds of sexual activities out of wedlock are not uncommon among both single and married college students, and among young men who work in Israel. Sexual activities mostly occur in cars, apartments, university dorms, and deserted locations.

“The proportion of youth engaging in different types of sexual activity is between 30% and 40%, and this is because we are a conservative society”. [17 year old woman, West Bank village, interview]

The majority of female participants indicated that young men engage in sex more than young women. They believe that young men have more freedom and think about sex more than young women. The fear of loss of virginity and bringing shame on their families are other factors inhibiting girls from engaging in sex before marriage.

“…the issue of virginity and honor reduces sexual practice by girls before marriage…” [19 year old woman, West Bank village, focus group]

“Young men have sex more than girls. Girls can control their desires; they are better than boys at controlling their emotions. Add to that, they get married at an early age. Boys mingle with the community and meet a lot of girls”. [23 year old woman, Jerusalem, focus group]

Many study participants reported that youth who live in cities tend to engage more in sexual activities than those who live in villages and camps. Many reported that youth engaged in sexual activities to a greater extent in the city of Ramallah, partly because it is a more liberal city than others in the West Bank and youth from surrounding areas come to it to engage in this type of activity more freely. Other locations identified by respondents as having a high rate of sexual activity by youth included Jerusalem, overseas, plus brothels in Israel or operating clandestinely in the West Bank, including East Jerusalem.

“I have friends who go out with men. They are happy to have boyfriends and always talk about their lovers. They hang out in Ramallah or in Jerusalem, but do not dare to meet in the camp”. [18 year old girl, West Bank camp, interview]

“I know many unlicensed brothels and they are well-known. I believe that Ramallah is the place where most things like this happen; it is not necessary to be a resident of Ramallah, but it is just that Ramallah is the center where people meet.” [17 year old girl, West Bank village, focus group]

Vaginal intercourse outside marriage was perceived to be less common than oral and anal sex due to fear of the potential consequences; it was most common among married women, for whom there is no fear of losing virginity, and especially when the spouse is absent. Homosexuality was referred to in most focus groups, but was perceived as less common.

“Young men do not go for penetration as they fear the consequences, so they do everything with the girl, but not penetration. They do not want to get into trouble”. [19 year old woman, West Bank city, interview]

“Sex comprises mostly anal and oral sex so the girl will remain a virgin”. [22 year old man, West Bank city, interview]

“Married women have more sex outside of marriage than unmarried ones because there is no fear about honor”. [A young woman over the age of 20, Jerusalem, focus group]

Virtual sex (phone and internet sex) was also reported to be prevalent among Palestinian youth. Some engaged in virtual sex on Skype or on the phone. Some called hot lines for a fixed sum of money. Some young men accused young women of seducing them by the way they spoke on the phone.

“There are the hot lines… if you want a girl at night or during the day”. [23 year old man, Jerusalem, interview]

“I used to call girls on the phone and exchange erotic words. The phone call would last about 20 minutes, or sometimes two hours”. [19 year old man, West Bank city, interview]

“Using the internet, we could chat and the girl would show herself on her camera”. [17 year old man, West Bank city, focus group]

Many participants were not well-informed about condoms, especially girls. Some young men participating in the focus groups indicated that youths do not use condoms when engaging in sex, especially when unplanned, or because some do not know how to use it. Other participants indicated that youths sometimes use condoms if they go to brothels, where the use of a condom is mandatory.

“People use condoms only in brothels in Israel. AIDS cannot be transmitted in brothels because it is compulsory by law to use a condom”. [Young man below 20 years of age, Jerusalem, focus group]

“We do not know if young men use a condom or not. We do not know how to use that thing…” [17 year old man, West Bank village, focus group]

“Sometimes I have used a condom, and other times I did not. It is not as if it [condom] is a cell phone to carry it wherever I go”. [23 year old man, West Bank city, interview]

### Models explaining sexual behaviors among Palestinian Youth

Most participants claimed that youth engage in sexual activities out of frustration and because they want to challenge the conservative culture. Others believe that problems between married couples such as being unable to satisfy the spouse, or separation or divorce, lead to sex out of wedlock.

“…pressure from parents and ‘forbidden fruit is sweet’”. [Young woman aged over 18 years, Jerusalem, focus group]

“Men and women engage in sex as a release; they are going to suffocate. They are unemployed, and those in jobs are on very low salaries; they need some release. It helps them feel good”. [17 year old man, West Bank city, focus group]

Other explanations given for sexual activities out of wedlock included financial constraints or fear of rejection by the family, e.g., for having a different socioeconomic status. However, some participants indicated that sex outside marriage is to fulfill a human need and there is nothing wrong with it. Many boys, especially those below the age of 18, accused women of seducing them by the clothes they wear or the way they talk.

“Young men engage in sexual activities because they do not have enough money to marry…” [A young man over the age of 19, Jerusalem, focus group]

“Tempting clothing causes guys to engage in sex”. [17 year old man, West Bank village, focus group]

Many young women believe that young men take advantage of ignorant and naïve girls.

“Some men have sex with ignorant, naive girls who have a weak character”. [23 year old woman, East Jerusalem, focus group]

“I know of a 15 year old girl who was exploited by an old man. This happens because of ignorance, and lack of awareness and loss”. [21 year old woman, East Jerusalem, focus group]

Some participants believe that youth engage in sex out of boredom and to fill their free time. Others believe that youth engage in sex out of curiosity and to experience something new, while others claim that young men engage in sex to prove their manhood.

“Others engage in sex to fill their free time”. [23 year old woman, Jerusalem, interview]

“As a result of peer pressure, they want to take a girl out and have fun with her to prove to their friends that they are studs”. [19 year old woman, West Bank city, interview]

A number of young women blamed parents for not keeping an eye on their daughters and for giving them a lot of freedom, or of oppressing their daughters so that once they go to college, they abuse the freedom and engage in risky behaviors.

“It is a frequent problem. After years of oppression and no freedom, the girl is suddenly free, without parental authority, and you can imagine what happens. She will definitely abuse the freedom and do things contrary to her culture, religion, family values and principles”. [21 year old woman, Jerusalem, focus group]

A few girls indicated that girls engage in sex to please their boyfriend in case the boys dump them for another girl.

“Some young men pressure girls to do everything with them. If the girls refuse, they leave them, so the girls do whatever is asked of them to keep their boyfriends”. [19 year old woman, West Bank camp, interview]

### Perceived positive and negative consequences of engaging in sexual activities

The top three negative consequences of engaging in sexual activities (in order of frequency of referral) were: health problems or physical disease; social stigma or disruption to social relationships; and sadness, regret, or mental repercussions.

“Semen may transmit infection through vaginal or anal intercourse. These infections will be very harmful, so girls should be cautious”. [19 year old woman, West Bank camp, interview]

The top three positive consequences of sex (in order of frequency of referral) were: personal pleasure and relief of desire; novelty, excitement, and risk; and increased status or prestige of manhood and masculinity (men only).

“It is a matter of relieving stress. No one cares about their health since, under these conditions, they want to satisfy their desire or they will explode because girls are so attractive. Before I was arrested, I used to say that a man should have fun and enter into relationships with every girl to fulfill his manhood”. [19 year old man, West Bank city, interview]

### Perceived need for sex education

Most of the participants, both males and females, emphasized the need for sex education because it was lacking in schools or at home.

“When a boy is not told about sex by the family, he looks for information from the street and watches pornographic films. The parents play the primary role in this process and must provide their children with information at a young age”. [A young man aged over 19 years, West Bank city, focus group]

“Levels of rape and sexual harassment are high in our society because of the lack of sex education”. [A young woman aged over 20 years, West Bank city, focus group]

**“**Sexual repression and lack of sex education are some of the main reasons for the engagement of youth in sexual activities outside marriage”. [A young man aged over 20 years, West Bank city, focus group]

**“**I do not dare to talk about sex with my mother. The minute I ask a question, she slaps me on the face and accuses me of being insolent. Actually, this is the first time I have talked about sex without being slapped on the face”. [A 17 year old woman, West Bank village, focus group]

## Discussion

Based on the Palestinian Family Survey of 2010, almost 22% of the population in Palestine is aged between 15 and 24 years [12]. These young adults face crucial political, social, and economic changes that affect life choices and behaviors. Unemployment, political and economic instability, and exposure to modern media and the internet may counteract the effects of conservative values and contribute to risk behaviors among Palestinian youth. Palestinian society is basically patriarchal in structure and gender inequality is deep-rooted. Over the past ten years, a growing number of reports of sexual assaults in marriages and families, sexual harassment, and femicide (honor killing) have been documented by various studies in Palestine [15]. According to our study findings, most of the young women reported that they engage in premarital sexual behavior as a reaction to the societal oppression they face.

There was a surprising willingness by the study participants to discuss sexual behavior and participants expressed their need to be heard and understood without being judged. Many did not want the focus group to come to an end and asked if future meetings would take place. This highlights the need for parents to bridge the generation gap and to build trust between them and their children by listening to their children’s fears and concerns without being judgmental, albeit while maintaining cultural and religious values. It is also clear that youth-friendly services are required to provide counseling and sex education.

Participants perceived that youth, especially males, engage in various types of sexual activity out of wedlock. Most of the participants agreed that non-penetrative and virtual sex are the most common acts in which youth engage. Participants were inconsistent on the question of whether youth generally use condoms.

Based on the study findings, unprotected sex and sex with sex workers is an area for concern. Not all sex workers use condoms, even those working in brothels in Israel [16]. Although a policy of condom use exists in Israel, a study of 55 women working in brothels in Israel found that some sex workers reported that they would be willing to forgo a condom if offered sufficient financial incentive and if the person looked clean [16]. In the West Bank, trafficking and prostitution are considered to be illegal activities, but operate informally on a small scale rather than as a sophisticated organized activity like those in Israel [17]. We do not have any information about condom use in these conditions, which thrive mainly in urban areas, primarily Ramallah and Jerusalem [17].

Another area of concern is virtual sex, which was reported to be prevalent among Palestinian youth. Although virtual sex does not constitute a danger to the physical health of young people, it may still have detrimental effects in terms of addiction to internet sex, or loss of significant real life relationships with a spouse, parent or close friends. As the behavior escalates, online use becomes chronic and more ingrained, developing into a compulsive obsession. At this stage, life becomes uncontrollable for the addict and relationships or careers may be jeopardized because of the compulsive behavior [18].

Many participants indicated that youth are more likely to engage in oral sex and anal sex to evade the risk of pregnancy. However although anal and oral sex entail significantly less risk of STI transmission compared with vaginal sex, they remain a potential transmission route for oral, respiratory, and genital pathogens, including STIs and HIV [19]. A potential rise in STI transmission rates due to oral and anal sex is a real danger as it is engaged in more frequently than vaginal sex and with no use of barrier protection [19].

In Palestine, worries about sexual activities outside marriage are related to the relatively low awareness of potential health hazards and lack of trusted youth-friendly services. Services tailored to young people that could inform about STIs and other health risks, and encourage preventative behavior, remain poorly developed. Currently, HIV services are mostly targeted at married women as part of reproductive health care [20, 21], rather than at younger unmarried men and women.

Subsequent work will use a large representative sample to extend our understanding of the prevalence and patterns of sexual behaviors. Confirmation of these findings will indicate the need for national policy responses and interventions to address risk behaviors and their causes.

## Conclusions

This is one of the first studies to directly investigate sexual behaviors. It has the standard limitation of a non-probability sample, namely that the youth interviewed are not representative of Palestinian youth in the West Bank, including East Jerusalem. In contrast with the conservative social context of the occupied Palestinian territories (oPt), the findings suggest that sexual activities outside marriage may be more common than is currently assumed. That said, this study highlights the need to put sexual reproductive health on the national agenda and incorporate it into the programs of national institutions. Policy responses, comprehensive interventions, and preventive programs targeting youth of both genders are required. We cannot stress enough the need for sex education and sexual health promotion for young people (both females and males) in the MENA region. Since most young people in the MENA region attend school, it would be a missed opportunity not to provide appropriate information about reproductive and sexual health as part of the educational curriculum [3].
